# Digital narratives in person-centered care: a scoping review

**DOI:** 10.1093/geroni/igaf144

**Published:** 2025-12-13

**Authors:** Irene Rodríguez-Trejo, Alba Felpete, David Facal, Raúl Vaca-Bermejo, Cristina Lojo-Seoane

**Affiliations:** Department of Developmental and Educational Psychology, University of Santiago de Compostela, Santiago de Compostela, Spain; Envita Digital Solutions S.L., Vigo, Spain; Department of Developmental and Educational Psychology, University of Santiago de Compostela, Santiago de Compostela, Spain; Institute of Psychology (IPsiUS), University of Santiago de Compostela, Santiago de Compostela, Spain; Department of Developmental and Educational Psychology, University of Santiago de Compostela, Santiago de Compostela, Spain; Institute of Psychology (IPsiUS), University of Santiago de Compostela, Santiago de Compostela, Spain; Envita Digital Solutions S.L., Vigo, Spain; Department of Developmental and Educational Psychology, University of Santiago de Compostela, Santiago de Compostela, Spain; Institute of Psychology (IPsiUS), University of Santiago de Compostela, Santiago de Compostela, Spain

**Keywords:** Digital storytelling, Life story work, Narrative care, Digital tools, Well-being

## Abstract

**Background and Objectives:**

Narrative care, an essential component of person-centered care (PCC), individualized care that respects unique life stories and preferences, uses personal narratives to foster understanding, trust, and emotional well-being. Digital storytelling has emerged as a valuable tool to collect and preserve these narratives. This scoping review explored digital tools used to capture life stories of older adults, summarizing their benefits, limitations, and implications for PCC.

**Research Design and Methods:**

Studies addressing digital storytelling, life review, or life story work with older adults, published in English or Spanish, and reporting cognitive, psychological, social, or care-related outcomes were included. A systematic search was conducted in Web of Science, SCOPUS, PubMed, and PsycInfo in December 2024, identifying 615 records. After screening and full-text review, 21 studies were included. Data were extracted on study design, sample characteristics, digital tools, outcomes, and limitations. Digital resources were classified into short or long videos, digital albums/collages, digital books/stories, and other formats.

**Results:**

The review encompassed 1,551 participants (age_*M*_ = 75.86 years). Digital storytelling improved emotional well-being, preserved personal identity, fostered social connections, and stimulated cognitive functions. Short formats proved particularly suitable when cognitive or digital literacy limitations were present, whereas longer formats supported in-depth reflection and memory preservation. Albums and digital books enhanced intergenerational communication. Key limitations included technological barriers, varying digital literacy levels, and lack of methodological standardization.

**Discussion and Implications:**

Digital storytelling enhances PCC by embedding personal narratives into care, but successful implementation requires addressing digital inequalities and standardizing methodologies through interdisciplinary collaboration.

Innovation and Translational Significance: Person-centered care in aging often lacks practical, scalable tools to integrate personal narratives into daily care practices. This scoping review identified a range of digital storytelling formats that support emotional well-being, identity preservation, social connection, and cognitive stimulation in older adults. These findings highlight the potential of digital storytelling to enhance individualized care and intergenerational communication in aging services. In addition, although several challenges emerged as secondary observations rather than primary focus areas of the review, acknowledging them can help guide the design of future interventions aimed at improving care quality and meaningful engagement in long-term care settings.

## Background and objectives

The person-centered care (PCC) approach considers that long-term care must adapt to individual needs and preferences. Thus, PCC places the individual at the center of attention and respects their particular characteristics, life plans, values, and preferences, unlike the traditional model of assistance, which tends to institutionalize care (Kitson et al., 2013; Martínez, 2011). The PCC model enables adoption of a more individualized and ethical view of care, as it considers people’s values and wishes regarding decisions about the provision of care (Kitwood & Bredin, 1992; Martínez, 2013). It also considers individual physical, emotional, and social dimensions, thus enhancing the professionals’ understanding of the person and favoring the creation of a relationship based on mutual trust and respect (Paiva et al., 2019; Rutz et al., 2017). Understanding all of these dimensions and the use of effective communication form the basis of PCC and have direct benefits on the person’s health (Greene et al., 2012; Yadav et al., 2020). Evidence suggests that personalized care that places individuals at the center of attention (and that values and listens to them) increases satisfaction with the care, improves relationships with carers, and favors adherence to interventions (Chan et al., 2013; Milota et al., 2019).

In the context of PCC, narrative care can be considered a good tool for improving the quality of care provided. Narrative care recognizes that each individual has a unique story. This story is formed by the person’s experiences, beliefs, and values, which will affect their health and relationships (including their interactions with carers) (Milota et al., 2019; Villar & Serrat, 2017). Considering these narratives will strengthen trust and empathy, enabling carers to know the person better and to obtain information that will favor decision-making about the most appropriate and effective types of care (Villar & Serrat, 2017).

Establishing aspects of the life history of the person being cared for is a key element of narrative-based care. This enables carers to make care more person-centered, focusing not only on symptoms but also on recognizing and validating the individual’s identity and experiences (Martínez, 2022; McKeown et al., 2006). Previous studies of narrative-based care have shown that remembering and sharing personal stories helps to improve self-esteem and a sense of belonging and may reduce depressive symptoms in older persons with no known cognitive impairment (Pinquart & Forstmeier, 2012; Westerhof et al., 2017). In addition, the benefits are maintained in the case of dementia, where the participation of family members and carers is essential to constructing a complete narrative. People with changes in cognitive function may experience difficulty recounting their stories, and this collaborative approach enables them to preserve their identity and dignity by recognizing their past experiences within the care context (Heggestad & Slettebø, 2015; McKeown et al., 2010). In addition, narrative care can also help people receiving palliative care to make sense of their experiences and address existential concerns related to living with advanced age and the end of life, such as questions of meaning, legacy, and continuity. In summary, this approach contributes to psychosocial and emotional well-being, demonstrating that working with personal narratives is a valuable tool for preserving identity and improving the quality of life of people (Lai et al., 2018; Lloyd‐­Williams et al., 2017).

Despite the benefits, there are some challenges associated with narrative-based care. These challenges are mainly related to the method of implementation and include a lack of standardization, limited resources, and inadequate training (Doran, 2019). Thus, a wide range of approaches and techniques has been used to collect people’s stories, including interviews, collages, memory boxes, life history books, presentations, pamphlets, and digital methods (Thompson, 2011).

In this context, digitalization enables the search for new ways to integrate narratives into PCC and provides diverse tools such as digital storytelling platforms that allow life stories to be recorded and shared more efficiently. This facilitates individuals’ participation in their own care and favors new forms of personalization (Puto et al., 2022). Evaluating both the benefits and shortcomings of implementing digitalization in PCC is particularly important in order to improve the quality of care and the well-being of older people. The scoping review is an ideal tool for this type of study as it gathers evidence on the use of digital narrative tools—i.e., digital storytelling tools used to document, share, and preserve older adults’ personal life stories to support individualized care—while also identifying knowledge gaps and clarifying key concepts in a diverse, emerging, and rapidly evolving area that has scarcely been investigated to date (Munn et al., 2018; Tricco et al., 2018). The aim of this review was to provide an overview of studies that have assessed various digital resources used to capture older adults’ life stories and related personal aspects, including their experiences, values, and preferences. This review also aimed to analyze the potential impact of the methodology on narrative-based care and the well-being of the individuals. As the digitalization of narrative-based care is an emerging practice, the review also aimed to identify the associated limitations and challenges in order to guide future research aimed at optimizing the use of digital tools in PCC.

## Research design and methods

This scoping review was conducted according to the relevant PRISMA-P criteria (Tricco et al., 2018), and the protocol was registered with OSF (https://osf.io/ent8c). Search was conducted in December 2024 in the Web of Science, SCOPUS, PubMed, and PsycINFO databases.

The following keywords and Boolean operators were used to search the databases: (“life review” OR storytelling OR “life stories” OR “life story” OR “story of life” OR “autobiographical narrative” OR “narrative care” OR “narrative gerontology”) AND (aging OR ageing OR elderly OR “older adults”) AND (digital OR technologies). These keywords were restricted to the title and abstract. This strategy enabled us to identify relevant studies addressing the use of life narratives or life stories with older adults in the context of aging and the use of digital technologies.

The references were managed using the Mendeley software. The initial searches generated a total of 615 articles (WOS: 406, Scopus: 150, PubMed: 39, PsycINFO: 20), and 140 duplicates were eliminated. Inclusion and exclusion criteria were established with the aim of focusing on the study objectives and guaranteeing the relevance and quality of the selected studies. Thus, the selected articles had to meet the following inclusion criteria: approaches involving life reviews or the use of life stories in the context of narrative-based care and effects at the psychological or care level, focused on the study of older adults (+50), including analysis of the use of digital technologies in the context of working with life histories; publications available in Spanish and English. On the other hand, articles that met the following criteria were excluded: studies focusing on patients with psychiatric alterations or other serious illnesses (e.g., diabetes, cancer, HIV), or on evaluating technological knowledge or the usability of tools.

**Figure 1.**
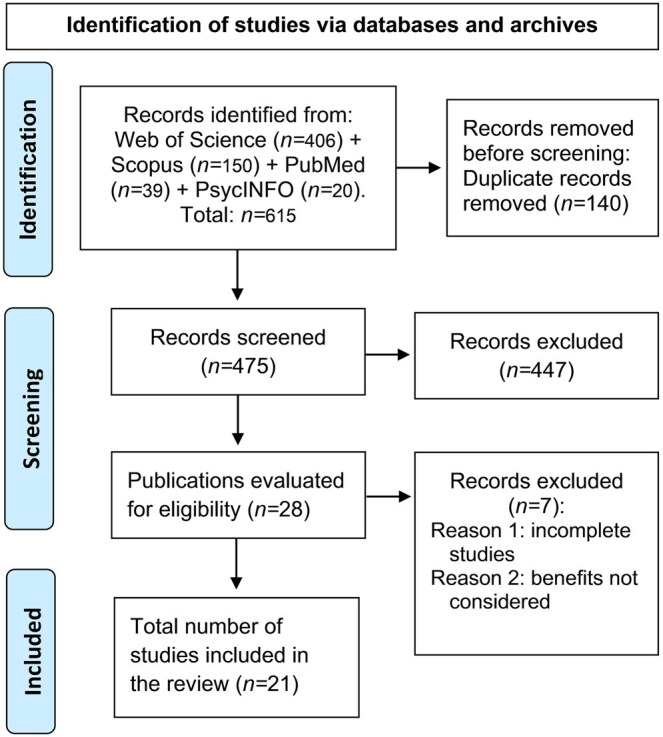
PRISMA flowchart.

An initial peer review was then carried out by I.R.-T. and A.F., who examined the title and abstract of the remaining 475 articles following the established inclusion and exclusion criteria. Any disagreements that arose during this process were resolved by consensus, resulting in the inclusion of a total of 28 articles. Finally, a full-text review was conducted. Seven of the articles were excluded as the studies were incomplete or did not meet the inclusion criteria (they did not consider the benefits to older adults). Thus, a total of 21 articles were finally included in the review. The process is summarized in Figure 1. The results of the analysis of the articles were categorized according to the digital resources used: “short videos,” “long videos,” “digital albums or collages,” “digital books or stories,” and “other resources.” For each resource, we systematically collected information on its characteristics, benefits, and limitations. In addition, the methodological limitations reported in the reviewed studies were recorded, which allowed us to take into account the methodological rigor of the research in this field.

## Results

During the review, we examined 21 studies that explored the use of digital narratives in older adults, with an accumulated sample of 1,551 participants. The mean age of the participants, calculated from the 11 articles that provided this information, was 75.86 years (Alexandrakis et al., 2020; Doi & Kuwahara, 2017; Elfrink et al., 2021; Gately et al., 2021; Keisari et al., 2022; Lind et al., 2024; Ríos et al., 2021; Smith et al., 2009; Subramaniam & Woods, 2016; Wang et al., 2024; Zhu et al., 2024). Regarding the sex distribution across participants, 69.55% were women and 30.45% men, although sex was not specified in seven of the articles (Chang et al., 2023; Crete-Nishihata et al., 2012; Dellkvist et al., 2024; Hausknecht et al., 2017; Li et al., 2020; Loe, 2013; Xu et al., 2023).

Data on participants’ cognitive status, the area of application of the method, and the persons involved are summarized in Table 1, while the main characteristics of the studies included in the review are presented in Table 2.

**Table 1.** Main characteristics of the samples in the reviewed studies.

Study	Cognitive impairment	Residential	Personnel involved
**Alexandrakis et al. (2020)**	No	No	Professionals and volunteers
**Chang et al. (2023)**	Both	Both	Professionals and volunteers
**Crete-Nishihata et al. (2012)**	Both	No	Family members
**Dellkvist et al. (2024)**	Not specified	Yes	Family and professionals
**Doi & Kuwahara (2017)**	Both	Yes	Professionals
**Elfrink et al. (2021)**	Both	No	Volunteers
**Gately et al. (2021)**	Both	No	Professionals
**Hausknecht et al. (2017)**	Not specified	No	Professionals and volunteers
**Hausknecht et al. (2019)**	No	No	Professionals
**Keisari et al. (2022)**	No	No	Professionals
**Li et al. (2020)**	No	Yes	Volunteers
**Lind et al. (2024)**	Both	Both	Family members
**Loe (2013)**	Not specified	No	Volunteers
**Ríos et al. (2021)**	Both	No	Professionals and volunteers
**Sehrawat et al. (2017)**	No	No	Volunteers
**Stargatt et al. (2022)**	Both	Not specified	Professionals and volunteers
**Smith et al. (2009)**	Both	Both	Family members
**Subramaniam & Woods (2016)**	Both	Yes	Professionals
**Wang et al. (2024)**	No	No	Professionals
**Xu et al. (2023)**	Not specified	Both	Professionals
**Zhu et al. (2024)**	Both	No	Professionals and volunteers

**Table 2.** Main characteristics of the reviewed studies.

Study	Main objective	Methodology	Sample	Digital resources	Results
**Alexandrakis et al., 2020**	To compare the impact of different media on the digital storytelling of older adults	Quasi-experimental mixed study	5 older adults (59–73 years) without CI	Various	The web-based platform reduced loneliness
**Chang et al., 2023**	To identify the psychosocial effects of digital storytelling in older adults	Literature review (19 articles)	360 older adults (50–99 years) with and without CI	Long videos	Improved mental health, community connectedness, digital literacy, and intellectual capacity
**Crete-Nishihata et al. (2012)**	To evaluate memory technologies in psychosocial well-being and autobiography	Quasi-experimental mixed study	12 older adults with Alzheimer’s disease or MCI and family members	Various	Promoted reminiscence and identity and reduced apathy
**Dellkvist et al. (2024)**	To explore the benefits and obstacles of using digitized life histories in dementia	Scoping review (31 articles)	Not specified	Various	Improved communication and social interaction and reduced challenging behavior.
**Doi & Kuwahara (2017)**	To evaluate a digital album in patients with dementia	Quasi-experimental qualitative (single case)	80-year-old woman with severe dementia	Digital album or collage	Reduction of behavioral symptoms and relational improvement
**Elfrink et al. (2021)**	To analyze the impact of digital reminiscence on mild dementia and caregivers	Randomized controlled trial	42 older adults with mild dementia and caregivers	Book or digital history	Modest effects on symptoms and caregiver quality of life
**Gately et al. (2021)**	To evaluate virtual narrative interviews in adults with CI	Observational study	14 older adults (77.5 years); various levels of CI	Book or digital history	High satisfaction of participants and caregivers
**Hausknecht et al. (2017)**	To evaluate a digital storytelling workshop for older adults	Quasi-experimental mixed study	40 older adults (>55 years)	Short videos	Improvements in digital storytelling, social collaboration, and technology
**Hausknecht et al. (2019)**	To evaluate the socioemotional impact of digital stories	Quasi-experimental qualitative	80 older adults (55–90+ years); no CI	Short videos	Increased social connection and satisfaction for the legacy created
**Keisari et al. (2022)**	To explore therapeutic experience with digital photocollage	Quasi-experimental qualitative	24 older adults (83.98 years); no CI	Digital album or collage	Artistic enjoyment, processing of life experiences
**Li et al. (2020)**	To evaluate a tangible device for the narration of memories	Qualitative study with interviews	6 couples (74–81 years old) and children	Tangible device	Promoted preservation of family history and generational connection
**Lind et al. (2024)**	To evaluate the impact of digital books on the identity of adults with memory difficulties	Experimental, quantitative	48 older adults (76.17 years) with CI	Book or digital history	Strengthened sense of identity and vividness of memories
**Loe (2013)**	To evaluate an intergenerational telephone digital storytelling project	Qualitative (no control group included)	Not specified	Short videos	Improved empathy, bonding, and understanding of personal history
**Ríos et al. (2021)**	To examine digital storytelling effects in older adults	Literature review (34 articles)	510 older adults (72.9 years); 52% with MCI	Short videos	Improved memory, confidence, identity, and social connection
**Sehrawat et al. (2017)**	Evaluate social connectedness in an intergenerational digital storytelling project	Quasi-experimental qualitative	4 older adults (73–82 years) + 4 students	Short videos	Increased social connection by sharing digital stories
**Smith et al. (2009)**	To evaluate multimedia biographies in Alzheimer’s disease	Qualitative exploratory (no control group included)	12 older adults (79.5 years old) with CI	Long videos	Promoted reminiscence, emotional well-being, and social interaction
**Stargatt et al. (2022)**	To review the impact of digital storytelling on health	Literature review (8 articles)	62 older adults (60–99 years old) with dementia or MCI	Short and long videos	Improved mood, memory, social connection, and quality of relationships
**Subramaniam & Woods (2016)**	To compare digital and conventional life books in dementia	Quasi-experimental single-case multiple	6 older adults (82.2 years) with mild/moderate dementia	Long videos	Improved quality of life, memory, and reduced depression
**Wang et al. (2024)**	To evaluate the usability of an AI-enabled photo album app for reminiscence.	Qualitative observational usability	13 older adults (65.8 years); no CI	Digital album or collage	92% positive experience, 85% facilitated recall
**Xu et al. (2023)**	To evaluate digital storytelling in reminiscence and the intergenerational component	Literature review (10 articles)	110 older adults (60 women, 50 men)	Various	Improved social connection, cognition, and emotional well-being
**Zhu et al. (2024)**	To co-design a digital storytelling intervention for MCI	Qualitative in three co-design phases	12 older adults (69.9 years old) with MCI	Digital application	Improved confidence and social interaction but required accessibility adjustments

*Note*. AI = artificial intelligence; CI = cognitive impairment; MCI = mild cognitive impairment.

### Digital resources

#### Short videos

The resources used to collect personal information included different digital formats. Most studies opted to use videos of various lengths and multimedia content related to the person’s life (e.g., presentations with photographs and music). Four studies involved producing short videos (Hausknecht et al., 2017, 2019; Loe, 2013; Sehrawat et al., 2017), and one study conducted a bibliographic review of these (Ríos et al., 2021). In the studies, short videos were understood as videos of maximum duration between 3 and 5 min, used to create personal life stories by combining images, music, narratives, and other multimedia elements (Hausknecht et al., 2017, 2019; Ríos et al., 2021; ­Sehrawat et al., 2017).

The articles reviewed highlight some of the observed benefits of using this type of resource, such as strengthening social ties, fostering a sense of belonging, and creating meaningful connections within the community (Hausknecht et al., 2017, 2019; Loe, 2013; Ríos et al., 2021; Sehrawat et al., 2017). They also emphasize that the process allows older adults to reflect on their life and reinterpret their memories in a positive light, providing a sense of accomplishment and meaning via the exchange of stories and thus contributing to the construction of a personal legacy (Hausknecht et al., 2019; Loe, 2013) and to improvement of emotional well-being (Hausknecht et al., 2019; Loe, 2013). The development of digital skills is another common benefit, which increases participants’ confidence and competence in using technology (Hausknecht et al., 2017; Sehrawat et al., 2017). Finally, producing short videos has been shown to be a useful tool for increasing awareness of healthy aging, providing participants with a way of reflecting on their life trajectory and promoting a positive image of aging (Ríos et al., 2021).

Some limitations were noted in studies using short videos as a resource. One of these is related to the older adults’ competence and interest in using the technology, which can influence the quality of the life stories created (Hausknecht et al., 2017; Loe, 2013).

#### Long videos

Some studies focused on the review and/or use of longer videos to present information about people’s life stories (Chang et al., 2023; Smith et al., 2009; Subramaniam & Woods, 2016). The duration of longer videos varied across studies, ranging from 12 to 27 min in Subramaniam and Woods (2016), whereas Smith et al. (2009) reported an average length of 39 min. Long videos are thus more extensive and detailed than short videos (Chang et al., 2023; Subramaniam & Woods, 2016). The context in which these videos were created also varied in the different studies. The videos were created from conventional life history books that were transformed into films, including personalized narration, music, and visual material compiled with the help of family members (Subramaniam & Woods, 2016), or personalized digital videos were created from information collected from the life histories of people with challenges or impairment or dementia (Smith et al., 2009).

The authors of all of the articles reviewed concurred that the use of longer videos contributes to improving the quality of life and well-being of the participants, reflected in increased self-esteem and a decrease in symptoms of depression, anxiety, restlessness, and apathy (Chang et al., 2023; Smith et al., 2009; Subramaniam & Woods, 2016), and helps to reduce problematic behavior in people showing signs of changes in cognitive function (Smith et al., 2009). The strengthening of autobiographical memory was another reported benefit, as the retelling and reflecting on life histories and exposure of participants to different stimuli helped them retrieve past memories and maintain their identity (Smith et al., 2009; Subramaniam & Woods, 2016). Communication was also facilitated, helping to create a greater number of social interactions between the participants, family members, and carers, which favored a better understanding of the life history of the older person (Chang et al., 2023; Subramaniam & Woods, 2016). Finally, the scoping review conducted by Chang et al. (2023) also highlighted that the use of longer videos contributed to improving participants’ digital literacy, particularly in terms of basic technical skills such as using tablets, computers, and multimedia applications.

However, some limitations were also reported in the studies. The main limitations identified were the availability of the resources required, in terms of both time and trained personnel (Subramaniam & Woods, 2016), and the lack of a standardized methodology for creating the multimedia content, which constrains comparison of similar studies (Chang et al., 2023). Smith et al. (2009) also indicated that the level of acceptance of the program may vary depending on the cognitive profiles of the participants, with use of this approach being of limited applicability in people with advanced dementia.

The review conducted by Stargatt et al. (2022) analyzes the impact of digital storytelling on the health of older adults with cognitive impairment (this review included both short and long videos [3 to 70 min]). These authors emphasized that both formats yield positive results, such as increased emotional well-being, improved self-esteem, and strengthened social connections with family members and carers, and that both short and long videos are valuable tools for working on reminiscence and for preserving personal identity (Stargatt et al., 2022). Although they did not directly compare the two formats, these authors found that long videos may enable a more in-depth exploration of the personal narrative, thus facilitating a greater sense of continuity and identity over time. However, short videos may be more accessible and are easier to produce, making them more appropriate for promoting participant involvement when resources are limited (Stargatt et al., 2022).

#### Digital albums and collages

Three of the studies used interactive digital albums or collages in which the participants actively selected and worked with photographs (Doi & Kuwahara, 2017; Keisari et al., 2022; Wang et al., 2024). Wang et al. (2024) described the development of the *GoodTimes* application, an interactive digital photographic album that enables users to explore their memories. Keisari et al. (2022) used a method in which participants selected and organized meaningful images from their lives to create visual compositions during sessions led by therapists. Doi & ­Kuwahara (2017) used interactive digital albums in residential centers, projecting images and videos during group sessions to encourage conversation among older adults, professionals, and family members, thus favoring a shared experience.

This methodology favors the exchange of memories by triggering conversations related to the images used. The results obtained in studies using this method show various benefits related to an increase in social connections and strengthening of relationships between the older adults, their families, and the carers (Doi & Kuwahara, 2017; Wang et al., 2024). The method also involves cognitive stimulation, specifically of autobiographical memory, as participants organize images and recall events that are important in their lives; this process can also support life review, in which these recalled experiences are reflected upon and reinterpreted in terms of their significance (Keisari et al., 2022; Wang et al., 2024). In addition, all of the studies report an increase in emotional well-being, thus generating greater personal satisfaction and reducing anxiety as a result of remembering significant moments of their lives (Doi & Kuwahara, 2017; Keisari et al., 2022; Wang et al., 2024). Finally, one of the studies reported a reduction in behavioral symptoms in participants with dementia (Doi & Kuwahara, 2017).

Regarding the limitations, the effectiveness of the interventions largely depended on the digital literacy of the participants and the continued support from family members or therapists to guarantee sustained participation (Keisari et al., 2022; Wang et al., 2024). In addition, the group sessions proposed by Keisari et al. (2022) may not be appropriate for all participants, especially those with advanced changes in cognitive function.

#### Digital books and histories

Three studies involved the creation of digital books or written histories, including information about different stages of people’s lives, complemented with multimedia content (Elfrink et al., 2021; Gately et al., 2021; Lind et al., 2024). Elfrink et al. (2021) proposed a methodology that allows participants to place memories on a dynamic timeline and to incorporate text and multimedia elements with the assistance of trained volunteers. Lind et al. (2024) described a reminiscence intervention based on autobiographical memory, in which structured interviews were used to help participants select key memories that are then organized in a personalized digital book. Finally, Gately et al. (2021) implemented the *My Life, My Story* program, an intervention that involved compiling the participant’s life story by conducting guided interviews, which are then transcribed and stored in the individual’s medical records.

All studies report improvements in social connections, as the intervention enables participants to share their memories with family members and carers, thereby promoting greater understanding of the person and strengthening family bonds (Elfrink et al., 2021; Gately et al., 2021; Lind et al., 2024). The researchers also reported that this type of intervention helps to preserve personal identity by helping the participants to create a coherent narrative, despite memory problems (Elfrink et al., 2021; Lind et al., 2024), and to reflect on their own lives, in addition to improving relationships with medical teams (Gately et al., 2021). Another observed benefit is a reduction in neuropsychiatric symptoms in people with mild changes in cognitive functioning, as well as a reduction in stress and caregiver burden, as access to the digital life stories facilitates communication and improves understanding of the needs of older people (Elfrink et al., 2021; Gately et al., 2021).

These studies have several limitations. The reliance of the intervention on technology hampers access for older people with low digital literacy (Elfrink et al., 2021; Gately et al. (2021), and professionals would have to be trained in methods of creating digital books, which could restrict large-scale implementation of the methods (Lind et al., 2024).

#### Other findings

Some of the studies reviewed used resources other than those mentioned above. For example, Li et al. (2020) used Slots-Memento, a device that combines photo viewing and audio recording and allows images to be selected via an interface resembling a slot machine. This resource helps participants recount their life stories and preserve memories of significant episodes in their lives, thus reinforcing identity and further stimulating autobiographical memory. The researchers also highlight that families may discover previously unknown stories, thus favoring relationships through intergenerational communication. The researchers describe the device as accessible and easy to use, highlighting the fact that the users do not need to be skilled in the use of digital technologies, an advantage for use by older adults, who tend to have a low level of digital literacy. However, the effectiveness of the process depends on the level of family involvement in preparing and supporting the storytelling, for example, by providing and digitizing personal materials, which may restrict its general use.

Zhu et al. (2024) used Huiyou, a digital resource, to collect information on the life stories of people with mild changes in cognitive function. The Huiyou application allows narratives to be created and exchanged. It can be used to retrieve memories, create narrative material, and share stories in group settings. Recounting and exchanging experiences with others via the application reinforces self-confidence, favors communication with family and friends, and stimulates memory and linguistic skills via the use of visual, auditory, and text stimuli. Use of the application was found to have beneficial effects related to social participation and the cognitive and emotional well-being of participants, thus contributing to reducing social isolation. Some limitations were also recognized, including difficulties related to usability, and the most recent version of the application, which has been improved on the basis of users’ suggestions, is currently being assessed. Future evaluation will therefore be required to validate the effectiveness of the application in natural contexts (Zhu et al., 2024).

Finally, other studies and literature reviews evaluated combinations of the previously mentioned resources, such as books and videos, as well as additional formats such as recordings, notebooks, and web pages (Alexandrakis et al., 2020; Crete-Nishihata et al., 2012; Dellkvist et al., 2024; Xu et al., 2023). Dellkvist et al. (2024) and Xu et al. (2023) conducted exploratory reviews to examine the use of digital narrative in older adults. Dellkvist et al. (2024) examined the use of digital life stories in the care of people with dementia, whereas Xu et al. (2023) considered the use of digital reminiscence in a sample of older adults and also evaluated intergenerational participation. Both studies concluded that digital narration facilitates communication and also contributes to preserving identity and improving the social and emotional well-being of older adults (Dellkvist et al., 2024; Xu et al., 2023). However, the researchers also highlighted some study limitations, such as the scarcity of time and resources in the care context, which prevented the successful use of digital life stories. The importance of accessibility was also highlighted as the use of these types of devices by older adults is often constrained by technological barriers (Dellkvist et al., 2024). Xu et al. (2023) suggested that further studies considering the intergenerational dimension and including a greater diversity of participants and methods are needed to improve the generalizability of findings.

Crete-Nishihata et al. (2012) evaluated different memory-targeted technologies among older adults with mild changes in cognitive function, using multimedia biographies, interactive screens located in the person’s surroundings and life-logging devices. These researchers found that all of the technologies evoked memories and favored family interactions, although some differences were noted: The biographies recorded on DVDs evoked more profound emotions, the interactive screens facilitated spontaneous interactions, and wearable life-logging devices such as SenseCam, which automatically capture daily images, improved episodic memory and reduced apathy. Some difficulties associated with usability, even of familiar technologies, were highlighted, and content production was found to be a time-consuming process, suggesting the need for more efficient tools for selecting and organizing materials.

Alexandrakis et al. (2020) compared the effect of collecting life histories in written format, with a voice recorder, and via a web platform. Relative to more traditional formats, the web platform proved to be the most effective resource for reducing loneliness and promoting social interaction between participants.

#### Limitations and research quality

Several methodological limitations were reported across the studies. A recurring issue was the small sample sizes, which limit the generalizability of the results (Alexandrakis et al., 2020; Hausknecht et al., 2017; Sehrawat et al., 2017; Zhu et al., 2024). Related to this, many studies were of short duration and lacked longer-term follow-up (Alexandrakis et al., 2020; Hausknecht et al., 2017; Sehrawat et al., 2017; Stargatt et al., 2022). In addition, the absence of control groups in several studies was reported in several studies (Hausknecht et al., 2017; Sehrawat et al., 2017; Stargatt et al., 2022).

Heterogeneity in study objectives and outcome measures was noted as another methodological limitation (Stargatt et al., 2022). Some studies relied primarily on qualitative interviews to evaluate participant well-being, which may limit the assessment of long-term effects (Gately et al. (2021; Li et al., 2020; Lind et al., 2024). In addition, potential bias in participant recruitment and selection was identified as a methodological concern in some studies (Gately et al., 2021; Lind et al., 2024).

## Discussion

In this review, we considered studies addressing different resources and digital formats used to collect information about older adults’ life stories. The findings indicate that compiling personal stories through digital resources may have positive effects on emotional well-being, personal identity, social relationships, and cognitive status in people with and without changes in cognitive function (Hausknecht et al., 2017; Keisari et al., 2022; Lind et al., 2024; Zhu et al., 2024).

### Digital tools and formats

Video recording was the most commonly used digital tool to compile the life stories in the reviewed studies. The use of this tool was classified according to the length of the videos produced, with short videos defined as those lasting between 3 and 5 min. The reviewed studies indicated that short videos contributed to individuals’ social (Hausknecht et al., 2017; Ríos et al., 2021; Sehrawat et al., 2017) and emotional well-being (Hausknecht et al., 2019; Loe, 2013). This type of video can be produced easily and quickly, facilitating its use in a wider range of situations for both groups and individuals (Hausknecht et al., 2017; Ríos et al., 2021; Sehrawat et al., 2017; Stargatt et al., 2022). However, brevity could also be considered a limitation, as the life stories were not presented in great depth, forcing individuals to select only brief fragments of their life experiences.

By contrast, longer videos had the advantage of enabling a more detailed look at participants’ life stories, providing greater narrative continuity and better integration. This format also tended to elicit a higher level of emotional involvement from participants recounting their experiences (Stargatt et al., 2022). This resource was particularly useful for families and caregivers of people with dementia, as it provided a space for sharing experiences (Smith et al., 2009; Stargatt et al., 2022; Subramaniam & Woods, 2016). The observed benefits included a reduction in depressive symptoms, strengthened autobiographical memory, and improved personal relationships, particularly through enhanced family bonds, intergenerational connections, and meaningful social interactions (Chang et al., 2023; Smith et al., 2009; Stargatt et al., 2022). Limitations included the time and resources required to produce this type of video, which may have represented a barrier for some potential participants (Alexandrakis et al., 2020; Subramaniam & Woods, 2016).

Other digital resources used to collect life stories included interactive digital albums and life history books. Studies using these types of resources showed positive effects on memory, emotional well-being and intergenerational communication (Doi & Kuwahara, 2017; Gately et al. (2021; Keisari et al., 2022), yet the positive effects of digital life story interventions appeared to depend on secondary factors such as participants’ level of digital literacy and the involvement of family members and care professionals (Lind et al., 2024; Wang et al., 2024). Although innovative tools such as digital apps (Zhu et al., 2024) and tangible devices (Li et al., 2020) were reported as accessible and easy to use, authors also noted important challenges, including limited generalizability of results and difficulties in sustaining the effects over time.

Several limitations were identified across the studies included in this review. These relate both to specific challenges associated with implementing digital life story tools and to broader conceptual and methodological issues that affect the overall quality and comparability of research in this field.

### Challenges in implementing digital life story tools

The studies reviewed highlight that the use of digital tools for collecting life story information presents a series of associated challenges that affect their effectiveness. Some secondary aspects that could favor or limit the use of these tools were identified, such as users’ digital literacy (Chang et al., 2023; Dellkvist et al., 2024; Elfrink et al., 2021; Gately et al. (2021; Hausknecht et al., 2017; Keisari et al., 2022; Loe, 2013; Wang et al., 2024), the cognitive profile of participants (Keisari et al., 2022; Smith et al., 2009; Stargatt et al., 2022) and the involvement of family members (Crete-Nishihata et al., 2012; Li et al., 2020; Subramaniam & Woods, 2016), the availability of resources (Crete-Nishihata et al., 2012; Dellkvist et al., 2024; Lind et al., 2024) and the usability of the tools (Crete-Nishihata et al., 2012; Elfrink et al., 2021; Gately et al. (2021).

#### Digital literacy

Digital literacy of participants was most frequently reported as a secondary factor influencing the use of digital tools. Lower digital competence limited the applicability of digital storytelling in older adults (Dellkvist et al., 2024) and reduced the quality and effectiveness of life stories produced through videos (Hausknecht et al., 2017; Loe, 2013), albums (Keisari et al., 2022; Wang et al., 2024), or books (Elfrink et al., 2021; Gately et al. (2021).

#### Cognitive profile

The cognitive functioning of users affected acceptance and limited the applicability of some tools, such as digital collages, for people with dementia.

#### Involvement of family members

The role of family members was essential for collecting audiovisual materials (Subramaniam & Woods, 2016) and for improving the effectiveness of some tools (Li et al., 2020). In addition, Crete-Nishihata et al. (2012) pointed out that digital memory tools can also have significant effects on family members and care professionals, suggesting that their experiences are inseparable from those of the older participants, indicating that the family context is not only a facilitator of the interventions but also an intrinsic part of how they unfold.

#### Availability of resources

The availability of resources such as time (Dellkvist et al., 2024), trained personnel (Lind et al., 2024), and efficient tools (Crete-Nishihata et al., 2012) for content production were also factors that influenced the use of these digital tools. Offering specific training to those involved in using the tools and having resources that streamline the organization and selection of materials can help save time in the production of digital content, which is where most of the time is spent.

#### Usability

Regarding usability, it is required to increase ease of use and accessibility to the technology and also to improve the digital training of older people. Such measures would also favor the generalization and efficacy of this type of intervention in the older population (Elfrink et al., 2021; Gately et al. (2021). Finally, collaboration between the designers and users of digital tools is required. Designers of this type of resource could benefit from considering the suggestions and opinions of the target users, their carers, and health professionals (Crete-Nishihata et al., 2012).

Beyond these practical and contextual challenges, there are also conceptual and methodological limitations that shape the quality of the evidence base in this field.

### Research quality and conceptual limitations

Regarding the quality of the studies analyzed, several methodological limitations were consistently reported. Small sample sizes and short intervention periods, often without long-term follow-up, restricted the generalizability of results and the ability to assess the sustained effects over time (Alexandrakis et al., 2020; Hausknecht et al., 2017; Sehrawat et al., 2017; Stargatt et al., 2022; Zhu et al., 2024). The absence of control groups in several cases further limited causal inferences, avoiding controlling for the possible influence of secondary variables, such as family involvement (Hausknecht et al., 2017; Sehrawat et al., 2017; Stargatt et al., 2022). Additional concerns included heterogeneity in study objectives, outcome measures, and methods of assessing effectiveness (Stargatt et al., 2022), which, in some cases, depended on qualitative interviews without complementary quantitative indicators. Quantitative measures would allow for more objective assessment of changes in cognitive and emotional well-being (Gately et al. (2021; Li et al., 2020; Lind et al., 2024). Another concern was the potential bias in participant recruitment and selection (Gately et al. (2021; Lind et al., 2024). Overall, these methodological limitations highlight the need for more rigorous and standardized research designs to strengthen the evidence base on the use of digital tools for collecting life story information in older adults.

A key limitation of our review process concerns the lack of conceptual consensus in the literature. The terms storytelling, life story work, life review, and narrative approaches have been used heterogeneously, sometimes as synonyms and at other times with distinct meanings. This variability posed challenges for both the search strategy and the synthesis of findings, potentially resulting in the inclusion of studies with partially divergent approaches. Developing shared definitions and theoretical frameworks could help reduce ambiguity, facilitate comparability across studies, and strengthen the knowledge base in this field.

### Practical implications and future research

The findings of this review highlight that digital storytelling can be a promising resource in older adults care, even in contexts with limited resources.

#### Tool selection

Selecting tools that are as simple and intuitive as possible, to offer training and guided support for their use (Hausknecht et al., 2017; Loe, 2013). Co-design approaches involving older people, families, and staff are particularly valuable, as they ensure that new tools reflect the real needs of users (Crete-Nishihata et al., 2012). Accordingly, institutions should not only offer training but also promote and participate in the development of technologies that are easy to adopt into daily care routines.

#### Cognitive adaptation

The selected digital tool should be suited to the cognitive profile of older adults or be flexible enough to adjust to their abilities. Short formats, such as collages or short videos, are particularly suitable when cognitive challenges are more advanced, and they also encourage participation and interaction (Hausknecht et al., 2017; Keisari et al., 2022). On the other hand, longer videos offer richer narratives and can serve as an important resource for families and staff in everyday care (Smith et al., 2009; Stargatt et al., 2022; Subramaniam & Woods, 2016). Using the appropriate resource, therefore, will be essential to optimize outcomes for people with diverse cognitive functioning. Although this review focused on analyzing the impact of digital stories in the context of the elderly population, it should be noted that the use of these digital tools could also benefit younger people in long-term or post-acute care situations, as they could facilitate continuity of identity and reinforce their connection with life experiences outside the institutional environment. In general, these methods could be relevant in other care settings where maintaining a sense of identity and personal meaning is beneficial, such as community programs for older adults or palliative care settings (Lai et al., 2018; Lloyd-Williams et al., 2017).

#### Family engagement

The reviewed evidence also highlights the role of families as a central element in the digital storytelling process, not only providing photos and basic information for the development of digital storytelling but also through their active participation in the narrative creation process, which enriches life stories (supporting memory among individuals with cognitive decline), strengthens intergenerational relationships (Keisari et al., 2022; Li et al., 2020; Subramaniam & Woods, 2016). Moreover, the creation of digital stories can provide families a sense of continuity and legacy, which can be helpful in coping with later stages of care or in the grieving process (Hausknecht et al., 2019; Loe, 2013). For institutions (nursing homes, social centers, etc.), it is valuable to promote structured opportunities, such as workshops or joint reminiscence sessions, where families and staff collaborate in the creation and review of digital narratives, transforming the intervention into a shared task rather than an individual one.

#### Resource optimization

Regarding resource availability, limited financial, human, and time resources can condition the implementation of these tools. Simple formats such as short videos and digital collages are affordable, practical, and capable of generating relevant results (Hausknecht et al., 2017; Ríos et al., 2021; Sehrawat et al., 2017; Stargatt et al., 2022). Volunteers, students, or family members (as highlighted above) can also provide valuable support (Elfrink et al., 2021). Developing tools that simplify material organization can further optimize production time (Crete-Nishihata et al., 2012). From a management perspective, implementation does not have to rely on large investments: pilot projects can be initiated with basic resources and gradually incorporate these digital practices into daily care routines.

#### Research recommendations

In research, methodological limitations remain a concern. Larger and more diverse samples, inclusion of control groups, and the use of standardized protocols are needed to improve comparability and replicability (Chang et al., 2023; Hausknecht et al., 2017; Stargatt et al., 2022; Subramaniam & Woods, 2016). Longitudinal designs are needed to assess whether the benefits of digital storytelling are sustained over time. Future research should also consider the heterogeneity of older adults (including intergenerational and cultural aspects) and combine qualitative and quantitative methods for a more comprehensive understanding (Gately et al., 2021; Li et al., 2020; Lind et al., 2024). Emerging technologies such as artificial intelligence (AI) may offer new ways to overcome barriers to digital literacy and support more accessible, personalized interventions in elder care.

#### Conceptual clarity

Finally, greater conceptual clarity and consensus regarding the terms *storytelling*, *life story work*, and *life review*. Although storytelling generally refers to a creative process of narrative expression, life story work is applied as a structured practice within care contexts, and life review is a reflective process focused on meaning-making across the life span. Clarifying these distinctions would facilitate comparability and theoretical coherence across studies.

In summary, the implementation of digital tools in elderly care settings is feasible, even in contexts with limited resources, through simple formats such as short videos or digital collages developed collaboratively with families, volunteers, and professionals. From a research perspective, more robust and standardized designs, larger and more diverse samples, and longitudinal studies are needed to evaluate the sustainability of benefits and the integration of new technologies such as AI.

## Conclusions

This review shows that the digital collection of life stories offers significant potential to enhance emotional well-being, personal identity, social relationships, and cognitive functioning in later life. Different formats, from short videos to digital books, offer flexible ways to tailor interventions to the cognitive profiles, needs, and contexts of older adults. At the same time, challenges related to digital literacy, cognitive functioning, family involvement, resources, and device usability. These factors, together with other methodological limitations, influence both the use and effectiveness of digital tools and should be carefully considered in future research aimed at strengthening the robustness of the evidence base.

Beyond these limitations, the findings provide practical guidance for the design, implementation, and accessibility of digital storytelling interventions in care settings, emphasizing the need to adapt tools to the specific characteristics of each population and context. As digital storytelling continues to evolve, it stands to benefit from the integration of co-design practices, ethical considerations, and emerging technologies such as AI. This integrated approach may enhance the relevance of digital life story interventions while promoting more personalized, inclusive, and sustainable practices in PCC.

## Data Availability

This scoping review is based on data extracted from publicly available scientific literature indexed in databases such as PubMed, Scopus, Web of Science, and PsycInfo. The data consist of bibliographic references and information charted from these articles, which are available to other researchers upon reasonable request. The protocol was registered with OSF (osf.io/ent8c).
